# Metabolic syndrome risk factors in overweight, obese, and extremely obese brazilian adolescents

**DOI:** 10.1186/1475-2891-12-19

**Published:** 2013-01-30

**Authors:** Anapaula CB Rizzo, Tamara BL Goldberg, Carla C Silva, Cilmery S Kurokawa, Helio RC Nunes, José E Corrente

**Affiliations:** 1 Department of Pediatrics, Botucatu School of Medicine, São Paulo State University (UNESP), São Paulo, Brazil; 2 Department of Pediatrics, Adolescent Medicine Course, Post Graduate Program in Gynecology, Obstetrics, and Mastology, Botucatu School of Medicine, São Paulo State University (UNESP), São Paulo, Brazil; 3 Department of Physical Education, University of North Paraná, Paraná, Brazil; 4 Clinical and Experimental Pediatric Research Center, Department of Pediatrics, Botucatu School of Medicine, São Paulo State University (UNESP), São Paulo, Brazil; 5 Statistical Consultant, Botucatu School of Medicine, São Paulo State University (UNESP), São Paulo, Brazil; 6 Department of Statistics, Botucatu School of Medicine, São Paulo State University (UNESP), São Paulo, Brazil

**Keywords:** Obesity, Metabolic Syndrome, Adolescents, Risk factors, Insulin resistance

## Abstract

**Background:**

Obesity in infancy and adolescence has acquired epidemic dimensions worldwide and is considered a risk factor for a number of disorders that can manifest at an early age, such as Metabolic Syndrome (MS). In this study, we evaluated overweight, obese, and extremely obese adolescents for the presence of MS, and studied the prevalence of single factors of the syndrome in this population.

**Methods:**

A total of 321 adolescents (174 females and 147 males) aged 10 to 16 years, attending the Adolescent Outpatient Clinic of Botucatu School of Medicine, Brazil, between April 2009 and April 2011 were enrolled in this study. Adolescents underwent anthropometric evaluation (weight, height, and abdominal circumference) and Body Mass Index (BMI) was estimated according to age and gender, following Disease Control and Prevention Centers recommendations (CDC, 2000). Blood pressure was measured and individuals with BMI ≥ 85^th^ percentile were submitted to laboratory evaluation for Total Cholesterol, HDL and LDL Cholesterol, Triglycerides, Fasting Insulinemia, and Fasting Glycemia to identify MS factors, according to the criteria suggested by the International Diabetes Federation. Insulin resistance was calculated by HOMA-IR, Quicki, and Fasting Glycemia/Fasting Insulinemia (FGI).

**Results and discussion:**

Of the 321 adolescents, 95 (29.6%) were overweight, 129 (40.2%) were obese, and 97 (30.2%) were extremely obese. Around 18% were diagnosed with MS. The most prevalent risk factors were abdominal circumference ≥90^th^ percentile (55%), HDL < 40 mg/dL (35.5%), High Pressure ≥130/85 mm/Hg (21%), Triglycerides ≥150 mg/dL (18.5%), and Fasting Glycemia ≥100 mg/dL (2%). Insulin resistance was observed in 65% of the adolescents.

**Conclusion:**

An increased prevalence of overweight and obesity, together with cardiometabolic risk factors such as dyslipidemia and abnormal blood pressure, were observed in adolescents, contributing to the onset of metabolic syndrome at younger ages. Risk factors for MS were more prevalent in females.

## Introduction

Obesity in infancy and adolescence has acquired epidemic proportions worldwide, with a high prevalence in both developed and developing countries [1]. It is considered a risk factor for a number of serious disorders, such as Metabolic Syndrome (MS), which can manifest at early ages. MS consists of a group of metabolic abnormalities, and according to the International Diabetes Federation (IDF), characteristics of this syndrome include obesity, with emphasis on excess abdominal fat, hypertension, dyslipidemia, and hyperglycemia [2]. Insulin resistance seems to be the physiopathological basis for MS and hyperinsulinemia is considered a precursor for MS. Weight gain is an independent predictor for MS development although not seen in all obese individuals [2].

Recently, the potential consequences of obesity and metabolic syndrome in adolescents have gained greater attention. Studies have shown that the factors of MS, which are abnormal in infancy, often persist throughout adulthood [2-4]. The prevalence of MS among adolescents in the United States has increased over recent years, from 9.2% in the period from 1988 to 1994, to 12.7% from 1999 to 2000 [5]. However, due to variations in the cutoff points adopted by different authors and a lack of consensus in defining the risk factors for MS, prevalence can vary according to the definition used and the population studied [2,3,6].

The criteria for diagnosing MS in children and adolescents have been established by the IDF [2]. They are specific according to age ranges between 6 and 10 years, between 10 and 16 years, and 16 years and over. It has been suggested that Metabolic Syndrome should not be diagnosed in children under 10 years old, but a reduction in body weight should be encouraged in those with central obesity. Between 10 and 16 years, MS can be confirmed by central obesity, defined using the 90^th^ percentile values of waist circumference for gender and age, associated to other two factors (elevated triglycerides, low HDL cholesterol, arterial hypertension, and hyperglycemia). Diagnosis criteria for adolescents of 16 years or above are similar to those for adults.

This study aimed to evaluate overweight, obese, and extremely obese adolescents for the presence of MS, and to establish the prevalence of each factor of the syndrome in the population analyzed.

## Materials and methods

Adolescents between 10 and 16 years old, of both genders, registered at the Adolescent Outpatient Clinic, Botucatu School of Medicine – UNESP, Brazil, between April 2009 and April 2011, were invited to participate in the study. Their parents or guardians signed an informed consent form. The study was approved by the Research Ethics Committee of Botucatu School of Medicine – UNESP, protocol 357/08 CEP. The age group was chosen following the criteria proposed by the IDF [2].

Participants underwent clinical examination, including anthropometric [7] and nutritional evaluation. Nutritional status was evaluated using Body Mass Index (BMI) curves, weight (kg)/height^2^ (m), according to age and gender, and the respective cutoff points proposed by the *Centers for Disease Control and Prevention*, which are: eutrophic between the 5^th^ and 85^th^ percentiles; overweight or, according to CDC [8] guidelines, “at risk of overweight,” greater than or equal to the 85^th^ percentile and less than the 95^th^ percentile; and obese, or according to CDC [8], “overweight,” above the 95^th^ percentile [8]. In this study, we used the terms overweight and obese. Participants with gender- and age-adjusted BMI above the 99^th^ percentile were classified as extremely obese [9]. Abdominal circumference (AbdC) was measured at the midpoint between the iliac crest and the last rib [9-11]. These values were compared with the 90^th^ percentile values of waist circumference according to age and gender, using the curve proposed by Fernández *et al.*[10]. Blood (BP), Systolic (SBP), and Diastolic (DBP) pressure were measured.

Participants considered overweight, obese, or extremely obese by BMI calculation (n = 321) were submitted to laboratory exams for total cholesterol and fractions, triglycerides, basal insulinemia (also called fasting insulinemia), fasting glycemia, free Thyroxinemia (free T4), and thyroid stimulation hormone (TSH), to evaluate the presence of diagnostic criteria for MS or another disease. Values published by IDF for this specific age group are Triglycerides (TG) ≥150 mg/dL, or specific treatment for this abnormality; HDL cholesterol (HDLc) <40 mg/dL for both sexes, or specific treatment for this abnormality; arterial hypertension with SBP ≥ 130 mmHg or DBP ≥ 85 mmHg or specific treatment for arterial hypertension; and fasting glycemia ≥100 mg/dL or a previous diagnosis of type 2 diabetes [2].

Fasting glycemia and fasting insulinemia values were used to calculate HOMA-IR (*Homeostasis model assessment of insulin resistance*), Quicki (*Quantitative insulin sensitivity check index*), and FGI (fasting glucose to insulin ratio). The cutoff points used to identify insulin resistance in participants were: fasting insulinemia >12μU/mL [12]; HOMA–IR > 3.16 [13,14]; Quicki <0.313 [15]; and FGI <7.0 [15].

The exclusion criteria were as follows: presence of metabolic, endocrine, or genetic disease as reported in patient records, detected by general and special physical examination, or in laboratory or radiological procedures, as well as menstrual cycle changes that indicate the presence of Polycystic Ovary Syndrome (PCOS) in female participants.

### Statistical analysis

Data for age, BMI, HDLc, TG, SBP and DBP, fasting Insulin and glycemia, HOMA-IR, Quicki, and FGI have asymmetric distribution. For comparison between gender-stratified groups, a generalized linear model (PROC GENMOD from SAS for Windows V9.2) with gamma distribution was used. Multiple comparisons were performed using the same program. AbdC data have symmetrical distribution and were analyzed using ANOVA followed by the Tukey test for gender-stratified groups. The level of significance adopted was 5%.

Associations between MS criteria, nutritional state, and BMI data for gender-stratified groups were analyzed through the Chi-square test or exact Fisher test. In all tests, significance level was fixed at 5% or the corresponding p value was used.

## Results

Out of 321 adolescents enrolled in this study, 95 (29.6%) were classified as overweight by BMI, 129 (40.2%) as obese, and 97 (31.2%) as extremely obese. Of these, 174 (54.2%) were female and 147 (45.8%) were male. Table 1 shows nutritional and laboratory results, and the criteria for MS diagnosis for both female and male participants.

**Table 1:** Nutritional assessment and laboratory variables for adolescents according to gender

**FEMALE**	**OVERWEIGHT (n = 55)**		**OBESE (n = 71)**		**EXTREMELY OBESE (n = 48)**	
	**X**	**SD**	**X**	**SD**	**X**	**SD**
**Age (years)**^(1) NS^	13.28^**a**^	2.26	12.66^**ab**^	2.05	12.45^**b**^	1.61
**BMI (kg/m**^**2**^**)**^(1)**^	25.10^**a**^	2.62	27.98^**b**^	3.07	33.99^**c**^	5.17
**AbdC (cm)**^(2)**^	82.84^**a**^	8.34	87.39^**b**^	8.85	95.84^**c**^	10.68
**HDLc (mg/dL)**^(1)**^	49.24^**a**^	9.95	42.19^**b**^	8.68	40.53^**c**^	13.08
**TG (mg/dL)**^(1)**^	92.11^a^	35.74	107.41^**b**^	68.11	121.33^**bc**^	46.38
**SBP**^(1)**^	110.73^**a**^	8.18	116.24^**b**^	14.53	127.58^**c**^	18.40
**DBP**^(1)**^	68.66^**a**^	6.89	71.47^**ab**^	12.63	81.93^**c**^	12.12
**Glycemia (mg/dL)**^(1)NS^	82.60^a^	8.43	83.58^a^	15.85	82.56^a^	7.52
**Insulin (**μ**U/mL)**^(1)**^	13.78^**a**^	4.79	17.58^**b**^	10.22	20.38^**bc**^	9.50
**HOMA-IR**^(1)**^	2.79^**a**^	0.99	3.65^**b**^	2.26	4.12^**bc**^	1.84
**Quicki**^(1)**^	0.33^**a**^	0.02	0.32^**b**^	0.02	0.32^**bc**^	0.02
**FGI**^(1)**^	7.37^**a**^	7.03	5.88^**b**^	2.69	5.12^**bc**^	3.18
**MALE**	**(n = 40)**		**(n = 58)**		**(n = 49)**	
	**X**	**SD**	**X**	**SD**	**X**	**SD**
**Age (years)**^(1)NS^	12.62 ^a^	1.76	12.89^**a**^	1.68	12.65 ^a^	2.22
**BMI (kg/m**^**2**^**)**^(1)**^	24.07 ^**a**^	2.12	27.12^**b**^	2.69	32.13^**c**^	4.11
**AbdC (cm)**^(2)**^	81.03^**a**^	7.54	86.02^**ab**^	10.14	95.19^**c**^	11.28
**HDLc (mg/dL)**^(1)NS^	43.10^**a**^	8.31	44.22^**a**^	11.37	44.31^**a**^	8.97
**TG (mg/dL)**^(1)NS^	103.38^**a**^	76.60	95.71^**a**b^	58.68	117.83^**ac**^	55.34
**SBP**^(1)**^	111.17^**a**^	15.01	115.16^**ab**^	14.10	125.98^**c**^	13.06
**DBP**^(1)**^	69.28^**a**^	10.70	74.73^**bc**^	10.20	76.88^**c**^	12.17
**Glycemia (mg/dL)**^(1)NS^	83.59^a^	7.22	83.77^a^	7.90	86.51^a^	5.79
**Insulin (**μ**U/mL)**^(1)NS^	13.72^**a**^	9.90	14.08^**a**^	7.64	16.13^a^	8.07
**HOMA-IR**^(1)NS^	2.84^**a**^	2.03	2.89^**a**^	1.62	3.45^**a**^	1.71
**Quicki**^(1)**^	0.34^a^	0.04	0.33^**ab**^	0.03	0.32^**b**^	0.02
**FGI**^(1)**^	10.86^**a**^	10.43	7.84^**b**^	5.99	6.32^**bc**^	2.38

**Note: **(1) Multiple comparison using PROC GENMOD from SAS program.

(2) Multiple comparisons using ANOVA and Tukey test.

* p < 0.05 **p < 0.01 NS not significant.

The same letters show no statistically significant difference.

Total number of female adolescents = **174.**

Total number of male adolescents = **147.**

*BMI *Body Mass Index, *AbdC *Abdominal circumference, HDLc-HDL cholesterol, *TG*Triglycerides, *SBP *Systolic Blood Pressure, *DBP* Diastolic Blood Pressure, *HOMA-IR *Homeostasis model assessment of insulin resistance *Quicki *Quantitative insulin sensitivity check index, *FGI *Fasting glucose to insulin ratio.

Regarding age, no significant differences were observed in comparisons between overweight, obese, and extremely obese groups for both male and female participants (Table 1).

Anthropometric values and MS criteria increased in direct proportion from the overweight to extremely obese female adolescent groups. Differences were statistically significant with the exception of glycemia (Table 1). The highest HDLc values were found in the overweight girl group, and the lowest values in the extremely obese girl group (p < 0.0001).

Mean fasting insulinemia values significantly increased from the overweight to the extremely obese girl group (p < 0.0001). The same was observed for HOMA-IR, where values increased in direct proportion from the overweight to extremely obese girl groups (p < 0.0001). The inverse was observed for Quicki and FGI mean values.

For male adolescents, the variables considered as risk factors for MS increased in direct proportion from the overweight to the extremely obese groups. However, no significant differences were observed for triglycerides, glycemia, and HDLc values between groups analyzed, and for fasting insulin and HOMA-IR. Mean Quicki and FGI values decreased in direct proportion from the overweight to extremely obese groups (p = 0.0031 and p < 0.0001).

Table 2 shows the prevalence of MS factors, according to IDF criteria, in all overweight, obese, and extremely obese adolescent groups classified by BMI and stratified by gender. Altered abdominal circumference was the most prevalent factor (55%), followed by reduced HDLc levels (35%), elevated pressure (21%), and elevated TG levels (18.5%). According to BMI classification, AbdC was the most prevalent criteria occurring in 87% of adolescents considered extremely obese.

**Table 2:** Prevalence of Metabolic Syndrome factors in all overweight, obese, and extremely obese adolescents according to BMI and by gender

		**General (n = 321)**	**Overweight (n = 95)**	**Obese (n = 129)**	**Extremely obese (n = 97)**
**Total**	AbdC >90^th^P ^(1)**^	55.0%	30.4% ^**a**^	54.5% ^**b**^	87.0% ^**c**^
	TG ≥150 mg/dL^(1)*^	18.5%	15.9% ^**ab**^	13.0% ^**a**^	29.6% ^**b**^
	HDLc <40 mg/dL^(1)NS^	35.5%	29.0% ^**a**^	37.7%^**a**^	40.7%^**a**^
	SBP-DBP ≥130/85^(1)**^	21.0%	10.1% ^**a**^	18.2% ^**a**^	38.9% ^**b**^
	Glycemia ≥100 mg/dL^(2)NS^	2.0%	2.9% ^**a**^	1.3% ^**a**^	1.9% ^**a**^
		**(n = 174)**	**(n = 55)**	**(n = 71)**	**(n = 48)**
**Female**	AbdC >90^th^P ^(2)**^	67.0%	41.9% ^**a**^	65.0% ^**a**^	96.6% ^**b**^
	TG ≥150 mg/dL^(2)NS^	19.0%	12.9% ^**a**^	15.0% ^**a**^	31.0% ^**a**^
	HDLc <40 mg/dL^(1)*^	41.0%	22.6% ^**a**^	47.5% ^**ab**^	51.7% ^**b**^
	SBP-DBP ≥130/85^(2)**^	21.0%	9.7% ^**a**^	15.0% ^**a**^	41.4% ^**b**^
	Glycemia ≥100 mg/dL^(2)NS^	3.0%	3.2% ^**a**^	2.5% ^**a**^	3.4% ^**a**^
		**(n = 147)**	**(n = 40)**	**(n = 58)**	**(n = 49)**
**Male**	AbdC >90^th^P^(1)**^	43.0%	21.1% ^**a**^	43.2% ^**a**^	76.0% ^**b**^
	TG ≥150 mg/dL^(2)NS^	18.0%	18.4% ^**a**^	10.8% ^**a**^	28.0% ^**a**^
	HDLc <40 mg/dL^(1)NS^	30.0%	34.2% ^**a**^	27.0% ^**a**^	28.0% ^**a**^
	SBP-DBP ≥130/85^(2)*^	21.0%	10.5% ^**a**^	21.6% ^**ab**^	36.0% ^**b**^
	Glycemia ≥100 mg/dL^(2)NS^	1.0%	2.6% ^**a**^	0.0% ^**a**^	0.0% ^**a**^

**Note: **(1) Chi-square test.

(2) Fisher’s exact test.

* p < 0.05 **p < 0.01 NS not significant.

The same letters show no statistically significant difference.

*AbdC *Abdominal circumference, *HDLc-HDL *cholesterol, *TG* Triglycerides, *SBP *Systolic Blood Pressure, *DBP *Diastolic Blood Pressure.

In females, the most prevalent criterion was altered AbdC, which varied from 41.9% in the overweight group to 96.6% in the extremely obese group (p < 0.001), followed by decreased HDLc levels, varying from 22.6% in the overweight group to 51.7% in the extremely obese group (p = 0.040). Arterial hypertension was the third most common factor of MS, occurring in 9.7% of overweight and in 41.4% of extremely obese girls (p = 0.007).

In males, the most frequent MS factor was abnormal abdominal circumference, which varied from 21.1% in the overweight group to 76.0% in the extremely obese group (p < 0.001). Decreased HDLc was the second most prevalent, but with no significant difference between groups, this was followed by hypertension and high triglyceride levels. The prevalence of hypertension was significantly higher in extremely obese than in overweight boys (p = 0.057) (Table 2). For both genders, altered glycemia was the least prevalent MS factor in all groups (Table 2).

When MS was diagnosed following IDF criteria, considering the presence of at least three out of five metabolic abnormalities, we found that from the 321 adolescents evaluated, 59 (18.3%) were positive for MS, of whom 32 were female and 27 were male. By the Exact Fisher test, the highest MS percentages were observed in the extremely obese groups regardless of gender (41.7% females and 30.6% males), and differed significantly to the other groups (p < 0.001 for females and p < 0.0042 for males).

From the 321 adolescents evaluated, 65% presented insulin resistance. Of the different methods used to evaluate insulin resistance, fasting insulinemia and fasting glucose to insulin ratio (FGI) resulted in the highest percentages of insulin-resistant adolescents (Table 3).

**Table 3:** Percentage of insulin-resistant adolescents according to method

**Method and reference value**	**General (n = 321)**	**Females (n = 174)**	**Males (n = 147)**
Insulin ≥12μU/mL	64.0%	74.0%	54.0%
HOMA >3.16	43.5%	54.0%	33.0%
Quicki <0.313	25.0%	32.0%	18.0%
FGI <7.0	65.0%	75.0%	55.0%

## Discussion

The prevalence of metabolic syndrome in obese children and adolescents has increased worldwide. Obese children have higher abdominal fat, which is associated with hyperinsulinism and cardiometabolic alterations such as low HDLc, increased triglycerides and LDLc, and increased blood pressure, resulting in increased risk for type 2 diabetes and cardiovascular diseases [6,9,16-19].

Our results are from a transversal study performed at initial clinical and nutritional evaluation of adolescents when they spontaneously signed up for consultation at the Adolescent Medicine Outpatient Clinic, which sees adolescents between 10 and 20 years of age and where consultations are arranged in advance [where the only limiting factor is the availability of professionals in this area to provide consultations]. In the study period, 321 adolescents between 10 and 16 years of age who presented with excess weight were included in the sample. We stress again that the participants are from a sample constructed for convenience, however those making up the sample were sequentially introduced, a measure incorporated into the treatment provided at the outpatient clinic. Therefore our results should be used with caution in overweight, obese, and extremely obese adolescents from other populations.

We found a high prevalence of overweight, obese, and extremely obese adolescents, together with a high prevalence of cardiometabolic risk factors, such as dyslipidemia and blood pressure alterations, in these individuals, potentially contributing to MS onset at early ages. The high prevalence of obesity found in this study clearly reflects the process of nutritional transition occurring in Brazil, through which undernutrition is replaced by obesity [20,21]. In a retrospective study of adolescents who also attended this clinic between 1988 and 1996, the authors showed that overweight and obesity doubled in females and more than tripled in males during this period [22]. Another study reported that the percentage of individuals who presented MS factors almost doubled over a ten-year period [19]

Anthropometric measurements, especially abdominal circumference, are crucial for MS diagnosis. In addition, measurements of serum lipid fractions, fasting glucose values, and blood pressure in susceptible or overweight individuals are also important. Evaluation of fasting insulin has been highlighted, given that a strong association between basal hyperinsulinemia, blood pressure alterations, and dyslipidemia has been demonstrated. Furthermore, insulin resistance has been indicated as the physiological basis for MS [23,24], since it precedes diabetes, anticipating insulin secretion failure [25]. It has also been suggested that hyperinsulinemia precedes the appearance of MS in infancy and adolescence, possibly explaining the association between obesity and the observed vascular dysfunctions [18].

In this study, an abnormal abdominal circumference was the most prevalent anthropometric parameter for both male and female adolescents, being more frequent in extremely obese females (96.6%) than in extremely obese males (76%). Of the 321 adolescents analyzed, 31% had at least one cardiometabolic risk factor, 26% had at least two, and 25% did not present any of the risk factors. The prevalence of three or more risk factors was higher in the extremely obese groups of both genders, at 41.7% in females and 30.6% in males. A previous study performed in Bogalusa, USA, found that 26% of adolescents had at least one risk factor and 4% had at least three risk factors. In extremely obese adolescents, 34% of females and 32% of males had at least three risk factors [9].

We also observed that the metabolic abnormalities were more significant and frequent in individuals with higher BMI values, specifically in obese and extremely obese adolescents compared to those considered overweight. Altered HDLc was the most frequent of the cardiometabolic parameters, followed by abnormal blood pressure and triglycerides levels. When the metabolic abnormalities were analyzed in groups stratified by gender, we observed an increased prevalence in females. Approximately 50% of extremely obese females had low HDLc levels and 30% had increased triglycerides. In males, HDLc was the most frequent (30%) altered cardiometabolic parameter, followed by increased blood pressure (21%), the latter being the most prevalent abnormality found in extremely obese males, with a frequency of 36%.

When analyzing the variables associated with insulin resistance, we found significant differences in adolescent female groups for all criteria. A higher percentage of girls (75%), than boys (55%), presented insulin resistance according to FGI values. Moran *et al.*[26]; Barja *et al.*[27]; Jeffery *et al.*[28] support our findings, as they stress the influence of sexual dimorphism, which results from the earlier appearance of secondary sexual characteristics in girls compared to boys, external biological expression modulated by hormonal ebullience belonging to the puberty years. For Jeffery *et al.*, HOMA-IR levels were higher in girls than boys at all evaluated ages, from 7 to 14 years, even after adjustments, presenting their peak when the adolescents were found in Tanner stage 3 and 4, moments associated with peak height velocity [28]. Regardless of gender, the extremely obese group presented the highest degree of insulin resistance. Our data differ from a previous study in which no significant differences in fasting insulin values were observed among eutrophic, overweight, and obese groups [4], possibly because analyses were conducted according to gender. We would like to emphasize that the cutoff points used in this study for anthropometric and biochemical variables and for insulin resistance are those recommended by IDF [2] for the studied age band, found in related international scientific literature [13-15], stressing that specific values for the Brazilian adolescent population are still not available. We believe that these values will soon be available as a large population study called the ERICA study (Study of Cardiovascular Risks in Adolescents) is being developed.

Considering the IDF criteria for MS diagnosis [2], we showed that around 18% of the adolescents analyzed presented the syndrome, which was more prevalent in extremely obese individuals, especially females.

A study in India with 2640 adolescents of both genders produced similar results; in eutrophic and overweight/obese adolescents, abnormalities in triglycerides, HDLc, basal insulin, and insulin resistance levels evaluated by HOMA-IR were higher in females. HOMA-IR values were much higher in individuals presenting parameters indicative of MS. Altered cardiometabolic parameters, of which reduced HDLc and increased triglycerides were the most common, were more frequent in overweight adolescents. Increased abdominal circumference was found in approximately 86% of overweight/obese adolescents [11].

The transversal approach used in this study raises the question of instability in metabolic syndrome diagnosis. Considering the intense growth and puberty changes, a longitudinal follow-up of our study individuals would be necessary. Nevertheless, studies have shown that 85% of obese individuals in this age group remain obese in adulthood [4,9] and many are diagnosed with MS. Even though some of these patients do not present the syndrome in the following years, its diagnosis during childhood and adolescence reinforces the importance of implementing effective treatment at an early age. Preventive measures aimed at reducing the incidence of obesity and its consequences at younger ages should also be adopted.

## Competing interests

The authors declare that they have no competing interest.

## Authors’ contribution

ACBR performed the sample collection, nutritional assessments, processed the data and drafted the manuscript. TBLG designed the study, performed the sample collection, nutritional assessments and processed the data, analyzed data and drafted the manuscript. CCS and CSK helped in data interpretation and in drafting of the manuscript. HRCN and JEC conducted statistical analysis and drafted the manuscript. All authors have read and approved the final version.
